# Ohio College of Dental Surgery

**Published:** 1863-04

**Authors:** 


### OHIO COLLEGE OF DENTAL SURGERY.

This old Institution has renewed its age, and is now fresher and more vigorous than in the days of its youth. With Kentucky invaded, Cincinnati under martial law, in fullest sense of that term, the whole South-west bristling with bayonets, some firmness was required to begin a course of lectures; yet, with part of the faculty away in the army, immediately after the "siege of Cincinnati" was raised, the College went to work with energy and success. At the close of the session, its friends assembled, (not all of them,) re-orgnized the faculty, and placed the Institution again on a permanent footing. Its prospects are now brighter than ever; and, in many respects, it is in better working order than ever before. The members of the faculty all belong to Cincinnati, and are thus in the *right place.* But better still, *they are the right men.* Of Professors Taylor, H. R. Smith, and Taft, nothing need be said. Their record is made. But it may be possible that we are better acquainted with the others than most of our readers; and hence, we are happy to say that Prof. H. A. Smith is *now* where he belongs. He was appointed Professor of Anatomy, and he would have filled that place creditably had he been allowed the privilege; but on a few days' notice, he was transferred to the department of Chemistry. This was an ordeal too severe for any young man, but Prof. S. sustained himself so as to be appreciated in his *natural field*, and was permanently transferred to this department. We had *picked him out* as our successor, during the first session of his pupilage.

Prof. Geo. E. Jones is a medico-dental—no, a *dental-medical* man, an experienced teacher, an accurate dissector, a thorough microscopist—the man for the chair of anatomy.

The establishment of an adjunct professorship of operative and mechanical denistry was a good move; and the appointment of Dr. E. Collins to fill it was still better. Dr. C. is an old graduate of the College, and has a very extensive and thorough experience in all the departments of Dental science.

We were unable to attend the meeting of the College Association, and felt very badly about it. We never failed before; but if the late meeting is to be the rule, we can afford to stay away, for no meeting incumbered with our presence ever done so much good work.

Success, then, to the OHIO COLLEGE OF DENTAL SURGERY; and success to all its sister institutions, for they are all needed.

